# The value of pre-transplant coronary angiography findings in kidney transplant candidates at high risk for cardiovascular disease

**DOI:** 10.3389/frtra.2023.1304516

**Published:** 2023-11-23

**Authors:** Leela Morená, Ayman Al Jurdi, Eduardo Leal Adam, Rucháma Verhoeff, Ragnar Palsson, Guilherme Taborda Ribas, Frank Hullekes, Abraham Cohen Bucay, Nahel Elias, Leonardo. V. Riella

**Affiliations:** ^1^ Center for Transplantation Sciences, Massachusetts General Hospital, Boston, MA, United States; ^2^ Harvard Medical School, Boston, MA, United States; ^3^ Department of Medicine, Division of Nephrology, Massachusetts General Hospital, Boston, MA, United States; ^4^ Universidade Federal do Paraná, Curitiba, PR, Brazil; ^5^ Transplantation Unit, Department of Surgery, Massachusetts General Hospital, Boston, MA, United States

**Keywords:** post-operative complications, coronary angiography, cardiac outcomes, risk factors, kidney transplantation

## Abstract

**Introduction:**

Cardiovascular disease is a significant cause of mortality after kidney transplantation. Whether pre-transplant screening for coronary artery disease (CAD) in asymptomatic kidney transplant candidates (KTCs) is beneficial is unclear.

**Methods:**

We conducted a retrospective cohort study evaluating post-transplant cardiovascular events in 192 high-risk KTCs who underwent pre-transplant CAD evaluation. The study aimed to identify risk factors associated with finding severe CAD on pre-transplant angiography, and to assess the relationship between screening strategies and post-transplant cardiovascular events.

**Results:**

At five years post-transplant, cardiovascular events occurred in 23.9% of subjects. Prior CAD history and left ventricular ejection fraction (LVEF) < 50% were associated with higher odds of finding severe CAD on pre-transplant angiography. Severe CAD on angiography was associated with a higher risk of early cardiovascular events within six months of transplantation. However, coronary intervention in KTCs with severe CAD was not associated with lower rates of post-transplant cardiovascular events.

**Conclusion:**

Pre-transplant coronary angiography to identify severe CAD is of highest yield in KTCs with a history of CAD or an LVEF < 50%. Our findings indicate that the identification of severe CAD in KTCs has prognostic significance for the early post-transplant period. Optimization of medical therapy in these high-risk KTCs may improve post-transplant cardiovascular outcomes.

## 1. Introduction

End-stage kidney disease (ESKD) has a well-established association with premature atherosclerosis, cardiovascular morbidity, and mortality (1). The 2020 Annual Data Report of the United States Renal Data System (USRDS) showed that about half of all known deaths in patients on hemodialysis were due to cardiovascular disease (CVD) (2).

Kidney transplantation (KT) is the preferred treatment for patients with ESKD and is associated with a reduction in long-term cardiovascular risk compared to remaining on maintenance dialysis (3). Despite the cardiovascular risk reduction of KT, CVD remains the leading cause of death after KT, with myocardial infarction (MI) risk being highest in the first three months posttransplant (4). Therefore, cardiac screening using non-invasive and/or invasive tests in asymptomatic KT candidates (KTCs) has become common practice in transplant centers. However, optimal strategies for utilizing such tests in this setting and whether pre-transplant coronary artery revascularization reduces peri-operative risk of cardiovascular events remain unclear (5, 6).

Prior data have suggested that non-invasive CVD screening prior to KT may be of little value, especially in patients with diabetes or preexisting CVD (7). Furthermore, the use of screening coronary angiography for the prevention of post-transplant cardiovascular events in KTCs has also been questioned by several studies (8, 9). The benefits of revascularization of asymptomatic obstructive coronary lesions prior to transplant are uncertain since the most critical contributor to myocardial infarction in both nonoperative and perioperative scenarios is the rupture of nonobstructive plaques (10). Additionally, invasive management of stable coronary artery disease (CAD) and advanced kidney disease has been shown to not reduce the risk of death or nonfatal myocardial infarction compared to a conservative management strategy (11).

In this study, we evaluated risk factors for post-transplant cardiovascular events in KTCs who underwent pre-transplant invasive and non-invasive CVD evaluation.

## 2. Materials and methods

### 2.1. Study design and data acquisition

We conducted a retrospective cohort study of all adult (≥18 years old) KTCs who were at high risk for CVD and underwent KT between 2016 and 2019 at one of our two academic medical centers, which have different protocols for pre-transplant CVD evaluation. At one center, the protocol is for all high-risk KTCs to undergo coronary angiography prior to KT, whereas at the other center, the protocol is to undergo stress testing, and only those with findings of reversible ischemia then proceed to angiography. The screening protocol was followed at both centers unless the transplant physician thought that CAD screening was unnecessary, despite the patients meeting high-risk criteria.

Data were collected manually from patient charts and are reported in compliance with STROBE guidelines. The study was approved by the Mass General Brigham institutional review board (protocol number: 2019P002526). The clinical and research activities being reported are consistent with the Principles of the Declaration of Istanbul as outlined in the ‘Declaration of Istanbul on Organ Trafficking and Transplant Tourism. This manuscript adheres to the Declaration of Helsinki.

### 2.2. Risk stratification and definitions of cardiac evaluations, intervention, and outcomes

Patients were deemed to be at high risk for cardiovascular events based on the presence of (1) history of diabetes mellitus (DM) of any duration, history of CAD, left ventricle ejection fraction (LVEF) < 50%, or history of ventricular arrhythmia or (2) age ≥50 years in combination with at least three of the following risk factors: hypertension, hyperlipidemia, history of cigarette smoking or ≥1 year on dialysis.

For this study, coronary angiography performed within five years of transplantation was considered as part of the pre-transplant evaluation. Coronary intervention was defined as either percutaneous coronary intervention (PCI) or coronary artery bypass surgery (CABG) following angiography. Patients who had previously experienced an acute myocardial infarction (MI) or had undergone coronary angiography revealing coronary artery disease (CAD) more than five years prior to transplantation were categorized as individuals with a prior history of CAD.

Severe CAD on pre-transplant angiography was defined as ≥70% disease in ≥1 vessel or triple vessel disease, as ≥50% stenosis of at least three epicardial coronary arteries (12). The vessels analyzed were the right coronary artery (RCA), the left main coronary artery, the left circumflex artery (LCx), the left anterior descending artery (LAD), and/or major branches of each artery. The composite cardiovascular outcomes included cardiac death, sudden death with no identifiable cause, non-ST elevation and ST-elevation non-fatal myocardial infarction, new arrhythmias (atrial fibrillation, ventricular tachycardia or ventricular fibrillation), and strokes after transplantation.

### 2.3. Statistical analysis

The unpaired *t*-test or Mann–Whitney *U*-test was used to compare continuous variables between groups, while Pearson’s Chi-squared test or Fisher’s exact test was used to compare categorical variables. Kaplan–Meier curves were used to estimate the incidence of outcomes with differences between groups assessed using the log-rank test. Univariable Cox regression was performed to assess associations between variables and the incidence of the composite outcome, with variables that were statistically significant or clinically relevant included in the multivariable model. The multicollinearity of variables was assessed using the variance inflation factor. Univariable logistic regression was used to evaluate the six-month risk of developing the composite outcome. The level of statistical significance was set at *α* = 0.05. Statistical analysis was performed using SPSS v24, and figures were created using Python 3.9.12, Pandas 1.5.0, Lifelines 0.27.4, Matplotlib 3.6.1, and GraphPad Prism v9.1.2.

## 3. Results

### 3.1. Cohort characteristics

192 kidney transplant recipients (KTRs) met the study’s inclusion criteria, 137 from Massachusetts General Hospital and 55 from the Brigham and Women’s Hospital. The baseline characteristics of the cohort are shown in Table 1. Invasive evaluation for CAD with angiography was performed in 115 KTRs (55%), non-invasive evaluation for CAD using stress testing was performed in 152 KTRs (73%). Most characteristics were balanced between those who underwent coronary angiography and those who did not (Supplementary Table S1).

**Table 1:** Baseline characteristics of the entire cohort.

Characteristic	All patients (*N* = 192)
Age (years), mean ± SD	58.7 ± 11
Male gender, *n* (%)	123 (64)
Race, *n* (%)	
Asian participants	8 (4)
Black participants	36 (19)
Caucasians participants	134 (70)
Other participants	14 (7)
Cause of end-stage kidney disease, *n* (%)	
Diabetes mellitus	101 (53)
Glomerular disease	34 (18)
Genetic disorders	25 (13)
Hypertension	12 (6)
Other or unknown	20 (11)
Time on dialysis, months, median (IQR)	34 (13–56)
Comorbidities prior to transplantation, *n* (%)	
Hypertension	187 (97)
Diabetes mellitus	142 (74)
Hyperlipidemia	161 (84)
Obesity	84 (44)
Coronary artery disease	64 (33)
Smoking	87 (45)

In the group who underwent angiography (*n* = 115), 38% had either no CAD or only minimal irregularities, while 25% had severe CAD. Only 22 patients (19%) of those who underwent angiography had revascularization with either PCI (*n* = 10) or CABG (*n* = 12). The median time from angiography to transplant was 0.7 years (IQR 0.3–1.5). Coronary angiography findings are summarized in Table 2. For those in the coronary angiography group, 74 KTRs (64%) underwent stress test, of which 16% had an abnormal result prior to the coronary angiography test.

**Table 2:** Angiography findings.

Variable	Coronary angiography group (*N* = 115)
Plaque characteristics, *n* (%)	
No CAD or minimal irregularities	44 (38)
Occlusion ≤ 50%	22 (19)
Occlusion 50%–69%	19 (17)
Occlusion ≥ 70%	30 (26)
Type of vessel disease, *n* (%)	
One vessel disease	28 (24)
Two vessel disease	15 (13)
Three vessel disease	7 (6)
Severe CAD, *n* (%)	30 (25)

### 3.2. Rate of the composite outcome

The median follow-up for the entire cohort was 48.0 months (IQR 38.9–58.2). In the angiography group, the median follow-up was 45.8 months (39.1–56.8), and 51.7 months (38.9–63.5) in the control group.

**Figure 1:**
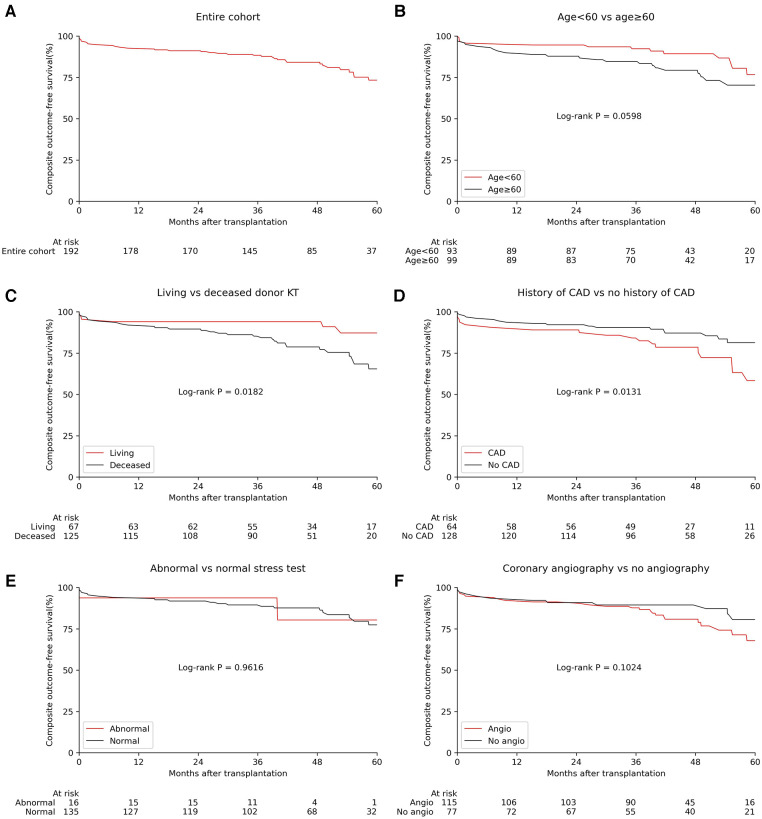
The incidence of the composite outcome stratified by pre-transplant risk factors, stress test and coronary angiography. (**B**–**F**) Statistics by Log-rank test. Angio, coronary angiography; CAD, coronary artery disease; KT, kidney transplant.

37 KTRs (19%) experienced at least one cardiovascular event during follow-up, of which eight occurred in the first 90 days after transplant, one between 3 and 6 months, four between 6 and 12 months, and twenty-four after the first year. The five-year Kaplan–Meier estimate for the incidence of the composite outcome was 23.9% for the entire cohort (Figure 1A). When stratified by coronary angiography prior to transplant, 26 KTRs (23%) in the angiography group developed the composite outcome during follow-up, compared with 11 KTRs (14%) from the non-angiography group. The median time-to-event in the angiography group was 32.6 (7.6–41.8) months, compared to 18.2 (3.8–54.4) months in the control group.

### 3.3. Risk factors for the composite outcome

We evaluated whether demographic and clinical characteristics were associated with a higher incidence of the composite outcome (Figure 1 and Supplementary Figure S1). We found that deceased donor kidney transplants (DDKT) and a prior history of CAD were associated with a higher incidence of the composite outcome (log-rank *P* = 0.018 and 0.013, respectively), while coronary angiography, sex, anginal symptoms, LVEF < 50%, DM and time on dialysis ≥6 months were not significantly associated with a higher incidence of the composite outcome (log-rank *P* > 0.05 for all). In those who underwent pre-transplant stress testing (*n* = 151), there was no significant difference between the incidence of the composite outcome between those who had normal vs. abnormal stress test results (log-rank *P* = 0.967).

**Figure 2:**
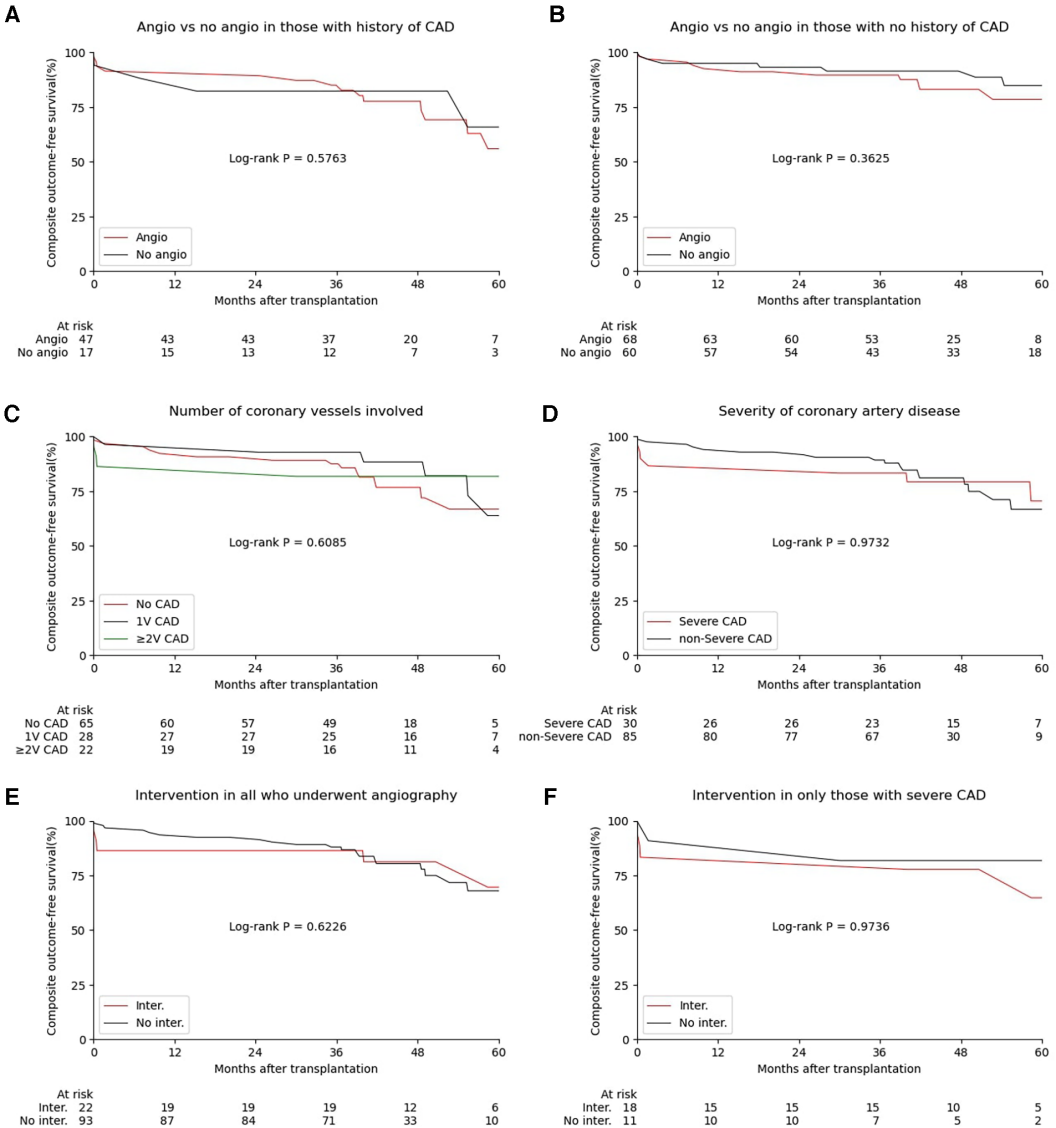
The incidence of the composite outcome stratified by pre-transplant coronary angiography, coronary angiography findings and interventions. (**A**–**F**) Statistics by Log-rank test. Angio, coronary angiography; CAD, coronary artery disease.

Since KTCs with known CAD may be more likely to undergo pre-transplant coronary angiography, we stratified the analysis for the association of pre-transplant coronary angiography with the composite outcome by history of CAD. We found no significant difference in the incidence of the composite outcome in those who underwent pre-transplant coronary angiography vs. those who did not when stratified by known history of CAD (log-rank *P* = 0.576, Figure 2A) versus the absence of a history of CAD (log-rank *P* = 0.362, Figure 2B).

In KTCs who underwent pre-transplant coronary angiography, we evaluated the association of angiography findings and interventions with the incidence of the composite outcome. When analyzing angiographic findings by the number of vessels involved (≥50% occlusion), there were several early events in those who had ≥2 vessel CAD, but in the long term, we found no significant difference in the incidence of the composite outcome in those with no CAD, 1 vessel or ≥2 vessel CAD (log-rank *P* = 0.609, Figure 2C). When analyzing angiography findings as severe CAD (at least one vessel with ≥70% occlusion or three vessels with ≥50% occlusion), there were also several early events in those who had severe CAD, but similarly, we found no significant difference in the incidence of the composite outcome in the long-term in those with or without severe CAD (log-rank *P* = 0.974, Figure 2D). Among those who underwent angiography, there was no difference in the incidence of the composite outcomes in those who subsequently underwent an intervention (PCI or CABG) compared to those who did not undergo an intervention (log-rank *P* = 0.623, Figure 2E). There was also no difference in the incidence of the composite outcome between those who did vs. did not undergo intervention in the subgroup with severe CAD (log-rank *P* = 0.974, Figure 2F).

**Figure 3:**
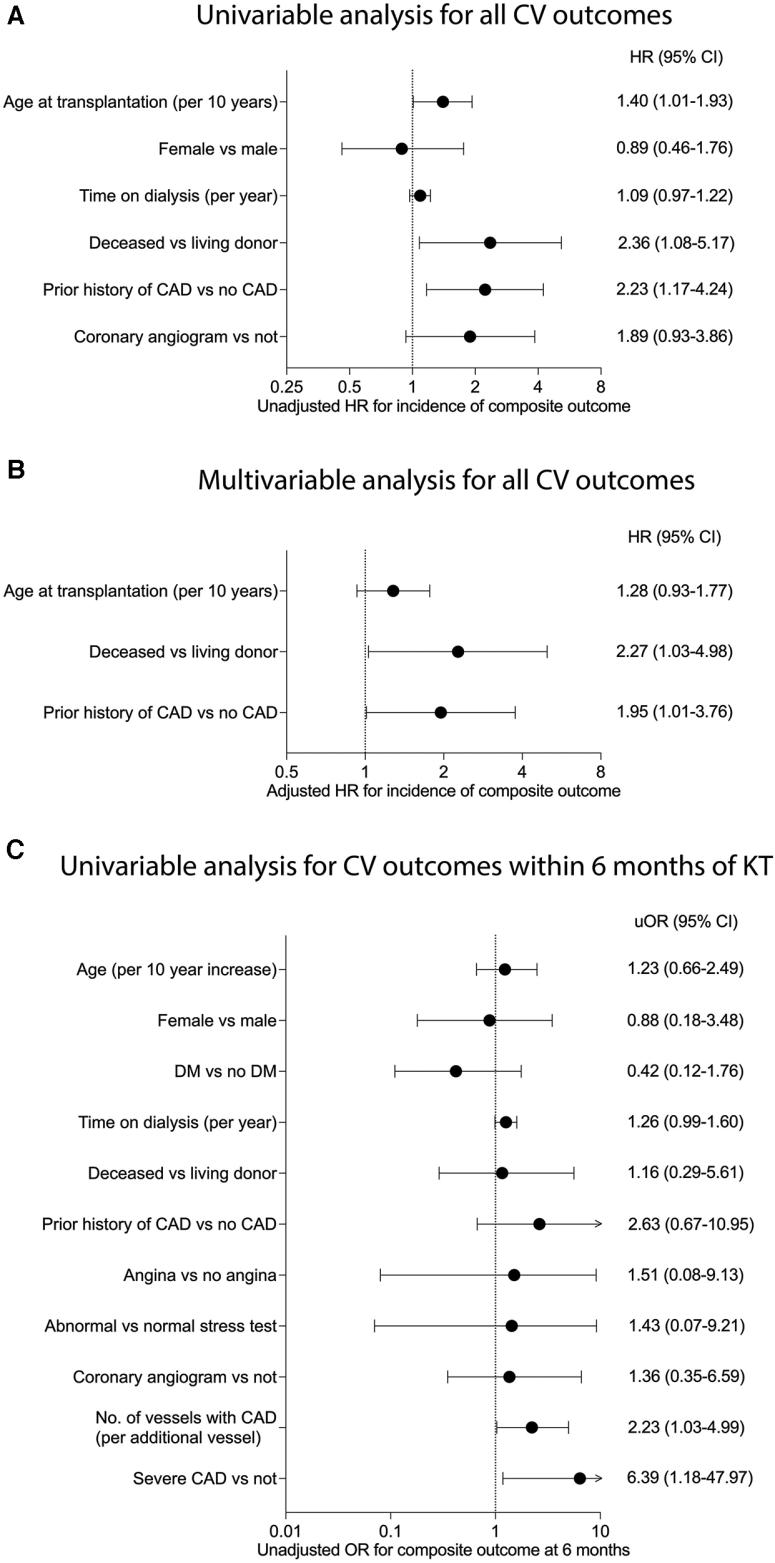
Risk factors for the development of cardiovascular events after kidney transplantation. (**A**) Unadjusted and (**B**) adjusted hazard ratios (HR) for the association between factors and the risk of development of the composite outcome (*n* = 192 for all). (**C**) Unadjusted odds ratio (OR) for the association between factors and the odds of development of the composite outcome within six months of kidney transplantation (*n* = 192 for all except for stress test [*n* = 151], 2 vessel CAD, severe CAD and intervention [*n* = 115], and intervention in severe CAD [*n* = 26]). Statistics by (**A**) univariable Cox regression, (**B**) multivariable Cox regression, and (**C**) univariable logistic regression.

For variables that met the proportional hazards assumption, univariable Cox regression was used to examine associations of baseline characteristics with the composite outcome (Figure 3A). Age (unadjusted HR 1.40 per 10-year increase, 95% CI: 1.01–1.93, *P* = 0.041), prior history of CAD (unadjusted HR 2.23, 95% CI: 1.16–4.24, *P* = 0.015), and DDKT (unadjusted HR 2.36, 95% CI: 1.08–5.17, *P* = 0.032) were associated with increased risk of developing the composite outcome (Figure 3A). There was no significant association between sex, time on dialysis, or pre-transplant coronary angiography with the risk of developing the composite outcome in univariable analysis (*P* > 0.05 for all). Using multivariable Cox regression to adjust for potential confounding variables, we found that DDKT (adjusted HR 2.27, 95% CI: 1.03–4.98, *P* = 0.041) and prior history of CAD (adjusted HR 1.95, 95% CI: 1.01–3.76, *P* = 0.047) were associated with a higher incidence of the composite outcome. Age (adjusted HR 1.28 per 10-year increase, 95% CI: 0.93–1.77, *P* = 0.132) was not associated with the development of the composite outcome in the multivariable model (Figure 3B). Pre-transplant coronary angiography was collinear with the history of CAD and, therefore, was not included in the multivariable model.

Since the risk of cardiovascular events conferred by pre-transplant risk factors may be higher in the early post-transplant period, we re-analyzed whether these factors were associated with the development of the composite within six months post-transplant using univariable logistic regression (Figure 3C). We found that a higher number of vessels with CAD (OR: 2.23 per additional vessel involved, 95% CI: 1.03–4.99) and the finding of severe CAD (OR: 6.39, 95% CI: 1.18–47.97) were associated with higher odds of developing the composite outcome within six months of transplantation. Revascularization in everyone who underwent angiography (OR 4.74, 95% CI: 0.82–27.36) and in the subgroup with severe CAD on angiography (OR 6.39, 95% CI 1.18–47.97) was not associated with the occurrence of the composite outcome within six months of transplantation. Multivariable logistic regression was not used to prevent model overfitting, as only a limited number of events occurred in the first six months.

### 3.4. Characteristics associated with severe CAD

**Figure 4:**
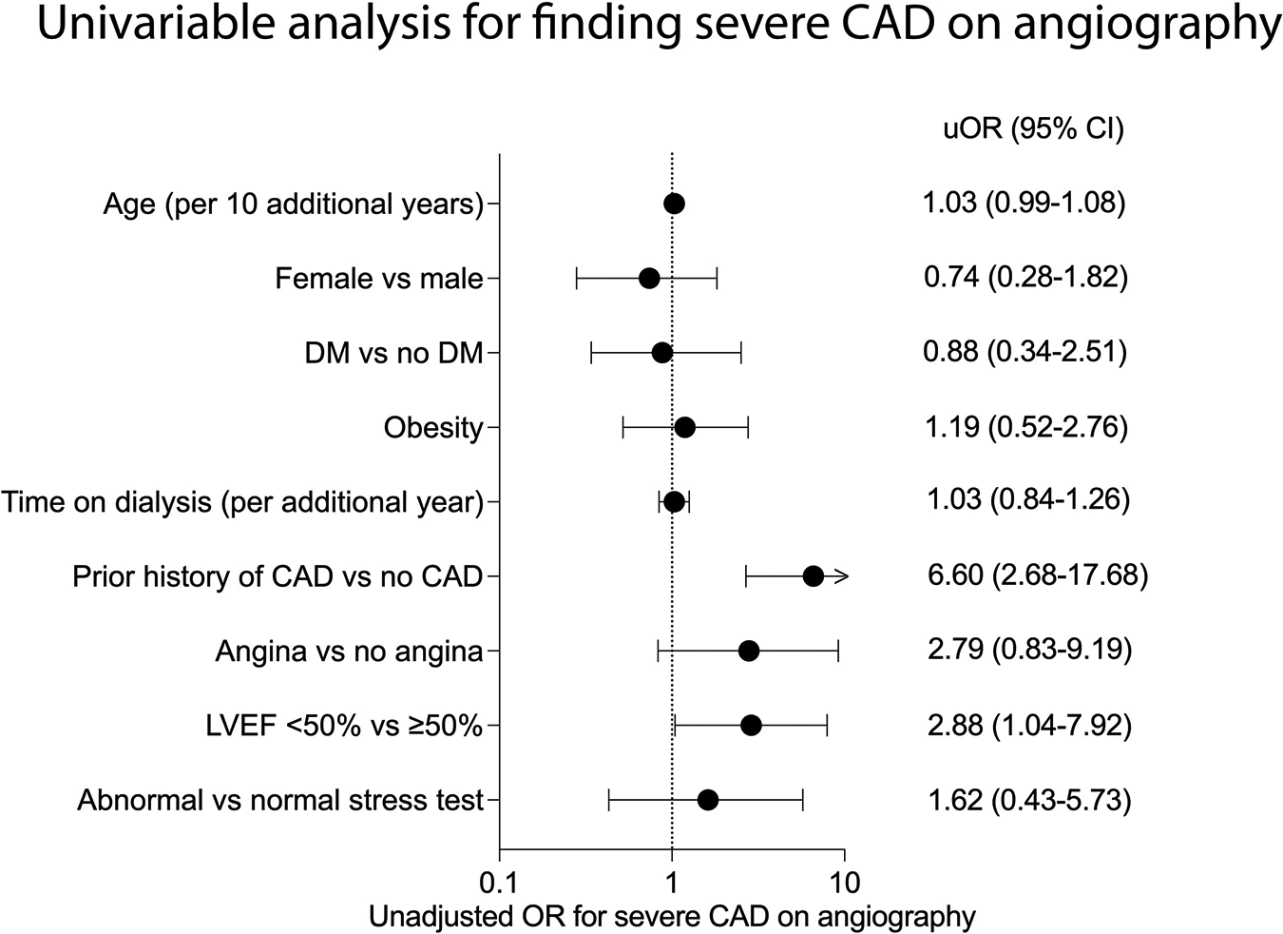
Factors associated with severe coronary artery disease on coronary angiography in kidney transplant candidates. Unadjusted odds ratios (OR) for the association between patient characteristics and finding severe coronary artery disease (CAD) on coronary angiography in kidney transplant candidates (*n* = 115 for all except for stress test, *n* = 74). Statistics by univariable logistic regression.

Since severe CAD was associated with a higher risk of developing the composite outcome within six months of transplantation, we then evaluated associations between baseline characteristics and the odds of finding severe CAD in the subgroup that underwent coronary angiography (*n* = 119). Univariable analysis showed that a history of prior CAD (OR: 6.60, 95% CI: 2.68–17.68) and LVEF < 50% (OR: 2.88, 95% CI: 1.04–7.92) before transplant were associated with higher odds of finding severe CAD on pre-transplant coronary angiography (Figure 4). Multivariable analysis was not performed to prevent model overfitting.

## 4. Discussion

In this study of KTCs, we evaluated risk factors associated with the finding of severe CAD on pre-transplant coronary angiography and the association of coronary angiography with the development of adverse cardiovascular outcomes after KT. Our main findings with regards to the diagnostic value of coronary angiography were that: (1) A prior history of CAD and LVEF < 50% were associated with higher odds of finding severe CAD on angiography, and (2) having severe CAD on pre-transplant angiography was associated with a higher risk of cardiovascular events in the first six months after KT. Taken together, these findings indicate that angiography may identify KTCs at high risk of cardiovascular events in the first 6 months after KT and is likely to be of highest yield in those with a prior history of CAD and with LVEF < 50%.

While angiography may be helpful for risk stratification, whether it is associated with an improvement in cardiovascular outcomes post-KT is not clear, especially given the low rate of intervention in our study and others (13, 14). Our study indicates that KTCs who underwent coronary angiography had a similar incidence of cardiovascular events post-KT compared to those who did not. To evaluate whether coronary interventions specifically were associated with improved cardiovascular outcomes, we compared cardiovascular outcomes in those who underwent coronary interventions compared to those who did not in two subgroups: (1) all those who underwent angiography, and (2) only those who had severe CAD on angiography. In both groups, we found no association between coronary intervention and the risk of developing adverse cardiovascular outcomes. However, our analysis has two limitations. First, it may have been biased by functionally more severe lesions being preferentially revascularized. Second, it was significantly underpowered to detect differences, especially in the subgroup of severe CAD. While our findings do not confirm a clear advantage of coronary interventions in preventing cardiovascular events after KT, the identification of severe CAD on angiography may prompt more aggressive medical management, including maximizing medications and lifestyle changes, which have been shown to be equally effective as an invasive intervention in patients with advanced kidney disease (5).

The efficacy of invasive screening for stable coronary artery disease in preventing cardiovascular events after surgery has been called into question. Studies in patients with or without kidney disease have already demonstrated no benefit of coronary revascularization for stable CAD prior to surgery. A retrospective registry study conducted on 2,572 KTRs from 18 different centers within the United Kingdom Renal Registry explored the practice of pre-transplant cardiovascular screening and its connection to post-transplant cardiovascular outcomes. In their study, 51% of the patients underwent CAD screening, either through stress tests or coronary angiography. By utilizing propensity score matching, the researchers determined that there was no discernible difference in the incidence of cardiovascular events at 90 days, 1 year, or 5 years post-transplant between KTRs who had undergone cardiovascular screening and those who had not. It is important to note that the registry study lacked detailed information, such as angiography findings and the performance of revascularization procedures, which precluded an assessment of the potential benefits of interventions in these patients. Furthermore, a recent registry study by Cheng et al. also suggested that pretransplant coronary angiograms for coronary heart disease were not linked to the composite outcome of death or acute myocardial infarction within 30 days following kidney transplantation (15).

Other studies have investigated the effectiveness of revascularization in improving outcomes for patients with stable CAD and kidney disease. A previous retrospective cohort study showed that coronary intervention in KTCs with ≥70% occlusion was associated with a trend towards improved coronary event-free survival compared to no intervention, but no difference in overall survival compared to medical therapy (5). Additionally, the ISCHEMIA-CKD study randomized 777 patients with advanced kidney disease (estimated glomerular filtration rate of <30 ml per minute per 1.73 m^2^ or on dialysis) and moderate-to-severe ischemia on non-invasive cardiac testing to undergo coronary angiography followed by revascularization (if necessary) or conservative medical therapy alone (11). The invasive strategy was associated with higher odds of stroke, death, or initiation on dialysis. Additionally, the secondary outcome (a composite of death, nonfatal MI, hospitalization for unstable angina, heart failure, or resuscitated cardiac arrest) was not significantly different between the groups. While this study does not answer the question of whether there is a benefit of pre-transplant cardiac angiography, it highlights the significant risks associated with coronary angiography and intervention in CKD patients (11).

Recently, the American Heart Association (AHA) released a scientific statement outlining an updated approach to screening for cardiovascular disease (CVD) in kidney transplant candidates (KTCs). (18) In their proposal, they introduced an algorithm for screening patients, distinguishing between those with a known history of coronary artery disease (CAD) and those without. For asymptomatic patients lacking a history of CAD, the AHA recommended considering coronary angiography only if a resting echocardiogram indicates a left ventricular ejection fraction (LVEF) below 40% or if there are regional wall motion abnormalities. In the case of symptomatic patients who do have a history of CAD, the AHA suggested referring them for a functional cardiac assessment, with stress echocardiography as the preferred method. Subsequent coronary angiography would only be advised if stress echocardiography reveals an LVEF below 40% or significant ischemia. Given that we found a higher risk of post-transplant cardiovascular events in KTCs with a history of CAD on univariable analysis, our study supports more thorough cardiovascular screening in this patient population. Regarding revascularization, the AHA’s scientific statement concluded that there is insufficient evidence to demonstrate the benefits of revascularization in asymptomatic KTCs (16).

Our study’s strengths include its multicenter design, the granular data collected, including coronary angiography findings and interventions obtained by individual patient chart review, and multivariable analysis for the incidence of the primary outcome to reduce bias from potential confounders. The limitations of our study include the small cohort size, which limits statistical power to find differences between groups (e.g., diabetes vs. not, smokers vs. not, abnormal stress test vs. not), and its observational retrospective design, which makes conclusions subject to bias from potential unidentified confounders, and limits our ability to draw firm conclusions about the benefits of coronary angiography and interventions on the incidence of cardiovascular events after KT. Lastly, our study did not collect data on medical therapy for non-intervenable coronary lesions.

Despite these limitations, we identified pre-transplant CAD and LVEF < 50% as risk factors associated with findings of severe CAD on coronary angiography, and severe CAD on angiography as a risk factor for early cardiovascular events post-transplant in KTC.

## Data Availability

The raw data supporting the conclusions of this article will be made available by the authors, without undue reservation.
