# Supplementary material for: The value of pre-transplant coronary angiography findings in kidney transplant candidates at high risk for cardiovascular disease
Source: Front Transplant. 2023 Nov 23;2:1304516. doi: 10.3389/frtra.2023.1304516 (PMC11235326; doi:10.3389/frtra.2023.1304516)
Supplement: Supplementary file 1 [file Datasheet1.pdf]

Supplemental Material

Table S1. Baseline characteristics in the cardiac angiography and control groups.

Characteristic	Cardiac angiography group (N=115)	Non-angiography group (N=77)	*P* value
Age (years), mean ± SD	59.4±9.9	57.6±12.3	0.291[a]
Male gender, n (%)	79 (69)	44 (57)	0.102[b]
Race, n (%)			
Asian participants	6 (5)	2 (3)	0.479[c]
Black participants	11 (9)	25 (32)	<0.0001[b]
Caucasian participants	86 (75)	48 (62)	0.065[b]
Other	12 (10)	2 (3)	0.048[c]
Cause of End-Stage Kidney disease, n (%)			
Diabetes mellitus	66 (57)	35 (45)	0.104[b]
Glomerular disease	18 (16)	16 (21)	0.361[b]
Genetic disorders	12 (10)	13 (17)	0.193[b]
Hypertension	7 (6)	5 (6)	0.999[c]
Other or unknown	12 (10)	8 (10)	0.999[c]
Time on dialysis, months, median (IQR)	35 (17-57)	31 (0-55)	0.145[d]
Comorbidities prior to transplantation, n (%)			
Hypertension	113 (98)	74 (96)	0.391[c]
Diabetes mellitus	90 (78)	52 (68)	0.097[b]
Hyperlipidemia	101 (86)	60 (77)	0.068[b]
Obesity	50 (43)	34 (44)	0.927[b]
Coronary artery disease	47 (41)	17 (22)	0.007[b]
Smoking	53 (46)	34 (44)	0.792[b]
Angina	13 (11)	2 (3)	0.029[c]
Median time of follow-up, months, median (IQR)	45.8 (39.1-56.8)	51.7 (38.9-63.5)	0.203[d]

[a] Statistics by unpaired t-test
[b] Statistics by Pearson's Chi-squared test.
[c] Fisher's exact test
[d] Statistics by Mann–Whitney *U*-test

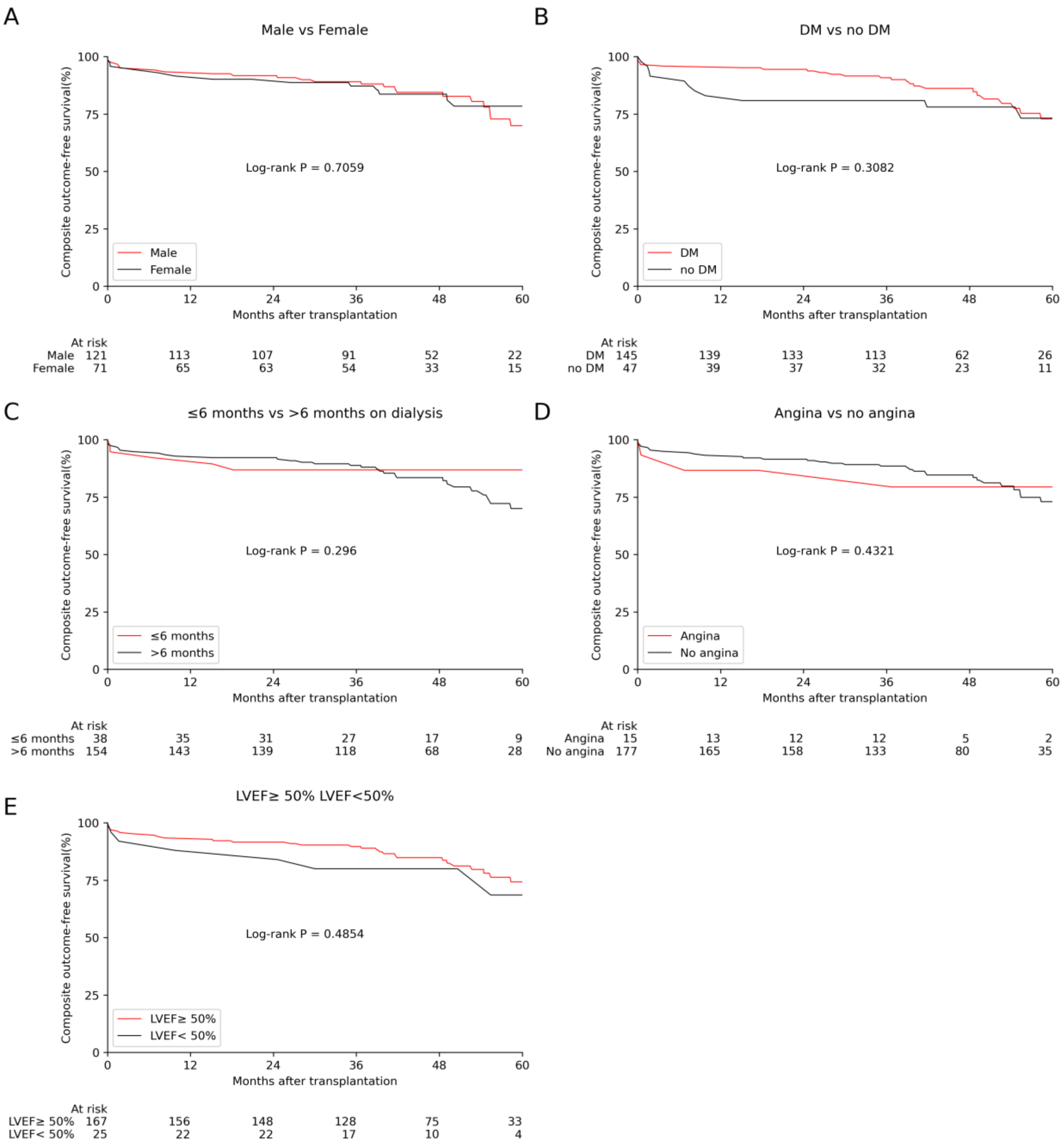

Figure S1. The incidence of the composite outcome stratified by pre-transplant risk factors, symptoms, and left ventricular ejection fraction (LVEF). (A-E) Statistics by Log-rank test.
